# Supplementary material for: Evolution of multifunctionality through a pleiotropic substitution in the innate immune protein S100A9
Source: eLife. 2020 Apr 7;9:e54100. doi: 10.7554/eLife.54100 (PMC7213983; doi:10.7554/eLife.54100)
Supplement: Supplementary file 1. [file elife-54100-supp1.docx]

**File S1. Alignment of modern and ancestrally reconstructed S100 proteins used in this study.**

>hA9_protein

------MTCKMSQLERNIETIINTFHQYSVKLGHPDTLNQGEFKELVRKDLQNFLKKENKNEKVIEHIMEDLDTNADKQLSFEEFIMLMARLTWASHEKMHEG--DEGPGHHHKPGLGEGTP-------------

>mA9_protein

-----MANKAPSQMERSITTIIDTFHQYSRKEGHPDTLSKKEFRQMVEAQLATFMKKEKRNEALINDIMEDLDTNQDNQLSFEECMMLMAKLIFACHEKLHENN-PRGHGHSHGKGCGK----------------

>opA9_protein

--------MENCTMEKALDIIVNTFHHYSTRVGNPDTLVKGEMKQLITKELPNFIKNA-KDLQDVKHLMQELDTNQNGQVDFKEFSMMMARLTMATHEKMHENA-PDKDHHSHGPGLEGKGGSSCGSGHGHGHSH

>ancA9_protein

----------MSQLEKAIETIINVFHQYSVRVGHPDTLNKKELKQLIQKELPNFLKNAKKDPQTINHLMQELDTNQDGQISFEEFMMLVARLTVASHEKMHENA-PEGDGHSHGPGLGGG---------------

>altancA9_protein

----------MSELEKSIETIINVFHQYSVRVGHPDTLSKGELKQLIQKELPNFLKNAKKDPQAIDHLFQDLDTNQDGQVSFEEFMVLVARLTVASHEKMHENA-PDKPHHSHGPGLEEK---------------

>hA8_protein

---------MLTELEKALNSIIDVYHKYSLIKGNFHAVYRDDLKKLLETECPQYIRK-----KGADVWFKELDINTDGAVNFQEFLILVIKMGVAAHKKSHEESHKE----------------------------

>mA8_protein

---------MPSELEKALSNLIDVYHNYSNIQGNHHALYKNDFKKMVTTECPQFVQN-----INIENLFRELDINSDNAINFEEFLAMVIKVGVASHKDSHKE--------------------------------

>opA8_protein

---------MATKLECAINCLVEVFHKYSLTGGHPHALSREQFGKLLEKECSEFTK---KSKKTVPEFMKELDINQDGFINFEEFLILTLKMVIEHHEDSHKE--------------------------------

>ancA8_protein

----------MTELEKAINSLIDVFHKYSLVAGHYHALSRDDLKKLLETECPQFLKKK-KDPKTVDTLFKELDVNKDGAINFEEFLILVTRVGVAAHEDIHKE--------------------------------

>altancA8_protein

----------MTELEKAINSLIDVFHKYSLVAGHYHALSKDDLKKLLEKECPEFMKKQ-KDPKTVDTFFKELDTNKDGQINFEEFLVLVTKVGVAAHEDIHKE--------------------------------

>hA12_protein

----------MTKLEEHLEGIVNIFHQYSVRKGHFDTLSKGELKQLLTKELANTIKNI-KDKAVIDEIFQGLDANQDEQVDFQEFISLVAIALKAAHYHTHKE--------------------------------

>chMRP126_protein

MSKGCQTQGPLSELEKAIDVIIDVFHQYSRREGDKDTLTRKELKLLIEKQLANYLKHV-KNQVSIDQIFKDLDNNKDQQLSFGEVMLLIIRVTVATHEHLHFCEDHQQQHQHQHQHQHNH---------------

>ancCG_protein

----------MSELEKAIETIINVFHQYSVRVGHPDTLSKKELKQLIQKELPNFLKNA-KDPATIDNLFQELDKNKDGQISFEEFMVLVARVTVACHEHMHKE--------------------------------

>altancCG_protein

----------MSQLEKSIETIINVFHQYSVRVGHPDTLSKGELKQLIQKELPNFLKNA-KDPATIDNLFQDLDTNKDGQVSFEEFMVLVARVTVASHEHIHKE--------------------------------
