# Supplementary material for: Evolution of multifunctionality through a pleiotropic substitution in the innate immune protein S100A9
Source: eLife. 2020 Apr 7;9:e54100. doi: 10.7554/eLife.54100 (PMC7213983; doi:10.7554/eLife.54100)
Supplement: Supplementary file 2. [file elife-54100-supp2.docx]

**File S2: Species-corrected S100 tree used for ancestral sequence reconstruction.**

TREE #1 with branch lengths:

(((S100B-Elephant_00000774: 0.011851, ((((S100B-Rat_00000790: 0.011435, S100B-Mouse_00000792: 0.000001): 0.000053, S100B-GuineaPig_00000756: 0.023169): 0.011662, S100B-Rabbit_00000772: 0.021407): 0.000104, ((S100B-Cow_00000764: 0.011306, S100B-Pig_00000782: 0.011385): 0.012895, (S100B-Dog_00000788: 0.257680, S100B-Cat_00000770: 0.010143): 0.000134): 0.000064): 0.012116): 0.094551, (S100B-Tasmaniandevil_00000794: 0.047966, S100B-Platypus_00000796: 0.156880): 0.056624): 0.023509, ((S100B-Turkey_00000800: 0.021217, S100B-Duck_00000808: 0.035275): 0.000090, S100B-Flycatcher_00000802: 0.012419): 0.018486, ((((S100G-Opossum_00000898: 0.161245, S100G-Tasmaniandevil_00000900: 0.073097): 0.072758, ((((S100G-Cow_00000862: 0.075218, S100G-Pig_00000864: 0.075304): 0.013547, (S100G-Cat_00000872: 0.028672, S100G-Dog_00000868: 0.058022): 0.000130): 0.013671, (((S100G-GuineaPig_00000876: 0.160619, (S100G-Rat_00000880: 0.012341, S100G-Mouse_00000886: 0.057520): 0.116953): 0.074398, S100G-Rabbit_00000878: 0.108386): 0.013349, S100G-Human_00000827: 0.044740): 0.000068): 0.007312, S100G-Elephant_00000892: 0.168194): 0.027686): 0.539077, (((((MRP-126-Greenseaturtle_00002085: 0.253840, ((((S100A8-Turkey_00000478: 0.072090, S100A8-Chicken_00000476: 0.000020): 0.000264, (S100A8-Duck_00000480: 0.109166, MRP-126-Goose_00002001: 0.010919): 0.041843): 0.074079, (MRP-126-EmperorPenguin_00002077: 0.186574, ((MRP-126-BarnOwl_00002030: 0.037136, MRP-126-Eagle_00002015: 0.035591): 0.010190, ((S100A8-Flycatcher_00000474: 0.020194, MRP-126-Zebrafinch_00002029: 0.031209): 0.093969, MRP-126-Budgerigar_00002048: 0.110229): 0.000062): 0.000087): 0.105688): 0.300170, (MRP-126-Alligator_00002081: 0.063287, MRP-126-Saltwatercrocodile_00002082: 0.079422): 0.125231): 0.081595): 0.076483, ((S100A12-Elephant_00000364: 0.375852, ((S100A12-Rabbit_00000366: 0.208679, (S100A12-Human_00000307: 0.000008, (S100A12-Gorilla_00000308: 0.011294, S100A12-Chimpanzee_00000310: 0.011316): 0.011289): 0.267716): 0.095317, ((S100A12-Cow_00000356: 0.132593, (S100A12-Sheep_00000358: 0.108573, S100A12-Pig_00000360: 0.126117): 0.028511): 0.033478, (S100A12-Cat_00000370: 0.142638, S100A12-Dog_00000338: 0.257857): 0.050457): 0.000517): 0.139973): 0.270007, (S100A9-Tasmaniandevil_00000558: 0.376463, S100A9-Opossum_00000560: 0.457951): 0.518003): 0.002864): 0.051883, (S100A8-Platypus_00000472: 0.118845, (((((S100A8-Human_00000391: 0.000001, S100A8-Gorilla_00000392: 0.023248): 0.176912, (S100A8-Rabbit_00000416: 0.187531, ((S100A8-Rat_00000424: 0.038358, S100A8-Mouse_00000428: 0.199436): 0.355484, S100A8-GuineaPig_00000436: 0.297343): 0.025972): 0.016180): 0.090034, ((S100A8-Cat_00000442: 0.071389, S100A8-Dog_00000452: 0.053183): 0.000277, (S100A8-Pig_00000456: 0.103224, (S100A8-Cow_00000438: 0.057455, S100A8-Sheep_00000448: 0.041907): 0.077606): 0.090118): 0.012641): 0.025245, S100A8-Elephant_00000462: 0.299468): 0.253959, (S100A8-Tasmaniandevil_00000468: 0.460050, S100A8-Opossum_00000470: 0.441062): 0.259349): 0.257176): 0.377056): 0.100813, ((S100A12-Opossum_00000380: 0.171391, S100A12-Tasmaniandevil_00000382: 0.287175): 0.326228, (((((S100A9-Sheep_00000534: 0.128256, S100A9-Cow_00000538: 0.045919): 0.046648, S100A9-Pig_00000542: 0.131808): 0.046797, (S100A9-Cat_00000540: 0.121027, S100A9-Dog_00000544: 0.023205): 0.114595): 0.090769, (((S100A9-GuineaPig_00000508: 0.315010, (S100A9-Mouse_00000514: 0.138646, S100A9-Rat_00000516: 0.120216): 0.182176): 0.113753, S100A9-Rabbit_00000506: 0.303617): 0.026172, (S100A9-Human_00000483: 0.009750, S100A9-Chimpanzee_00000486: 0.000001): 0.194039): 0.024606): 0.000327, S100A9-Elephant_00000526: 0.263248): 0.273405): 0.171144): 0.119685, ((((((S100A7-Eagle_00003007: 0.111865, S100A7-BarnOwl_00003010: 0.091265): 0.036718, (S100A7-Zebrafinch_00003027: 0.345523, S100A7-Budgerigar_00003002: 0.094486): 0.031823): 0.000200, S100A7-EmperorPenguin_00003023: 0.158408): 0.000450, ((S100A7-Goose_00003015: 0.025054, S100A7-Duck_00003021: 0.070377): 0.176006, S100A12-Turkey_00000386: 0.306748): 0.086695): 0.200304, (S100A7-Saltwatercrocodile_00003038: 0.208248, S100A7-Alligator_00003036: 0.149850): 0.259960): 0.646359, (((((S100A7-Cow_00000594: 0.063900, S100A7-Sheep_00000590: 0.126150): 0.166292, S100A7-Pig_00000608: 0.358581): 0.006962, (((S100A7-Chimpanzee_00000570: 0.022260, (S100A7-Human_00000569: 0.022139, (S100A7A-Chimpanzee_00000632: 0.010951, (S100A7A-Human_00000631: 0.045504, S100A7A-Gorilla_00000634: 0.010950): 0.000001): 0.034351): 0.000001): 0.161014, (S100A7L2-Human_00000701: 0.000076, S100A7L2-Chimpanzee_00000702: 0.011065): 0.571687): 0.109920, S100A7-Rabbit_00000622: 1.129756): 0.000404): 0.104943, S100A7L2-Elephant_00000722: 0.365579): 0.838659, (S100A7-Tasmaniandevil_00000628: 0.070559, S100A7-Opossum_00000630: 0.031288): 0.685999): 0.127986): 0.444082): 0.054121): 0.183817, (((((((CRNN-Pig_00000046: 0.021872, (CRNN-Sheep_00000058: 0.000012, CRNN-Cow_00000062: 0.012337): 0.049611): 0.053537, (CRNN-Cat_00000052: 0.062868, CRNN-Dog_00000044: 0.098552): 0.000113): 0.000129, ((((CRNN-Mouse_00000030: 0.011248, CRNN-Rat_00000036: 0.037055): 0.104929, CRNN-GuineaPig_00000024: 0.081970): 0.017533, CRNN-Rabbit_00000038: 0.101269): 0.000147, (CRNN-Chimpanzee_00000002: 0.023821, (CRNN-Human_00000001: 0.000001, CRNN-Gorilla_00000008: 0.000001): 0.000001): 0.062164): 0.000053): 0.013720, CRNN-Elephant_00000066: 0.046485): 0.164013, (CRNN-Opossum_00000072: 0.021379, CRNN-Tasmaniandevil_00000074: 0.039367): 0.053794): 0.344728, (CRNN-Duck_00000080: 0.077283, (CRNN-Turkey_00000076: 0.074076, CRNN-Chicken_00000078: 0.096950): 0.172464): 0.198220): 0.324597, (((((HRNR-Cat_00000210: 0.026197, HRNR-Pig_00000206: 0.099361): 0.028399, ((HRNR-Gorilla_00000182: 0.011289, (HRNR-Human_00000179: 0.000001, HRNR-Chimpanzee_00000180: 0.000001): 0.000001): 0.095248, (HRNR-GuineaPig_00000196: 0.117870, (HRNR-Rat_00000192: 0.071187, HRNR-Mouse_00000194: 0.048424): 0.129632): 0.017313): 0.020017): 0.267241, ((FLG-Platypus_00000108: 0.166845, (((((FLG2-Rabbit_00000132: 0.061346, ((FLG2-Rat_00000138: 0.012063, FLG2-Mouse_00000140: 0.036027): 0.067101, FLG2-GuineaPig_00000150: 0.145605): 0.008646): 0.010452, (FLG2-Human_00000117: 0.023588, FLG2-Chimpanzee_00000122: 0.000001): 0.048487): 0.000136, (((FLG2-Sheep_00000134: 0.011315, FLG2-Cow_00000136: 0.012165): 0.023989, FLG2-Pig_00000146: 0.011764): 0.000110, FLG2-Cat_00000148: 0.011761): 0.000095): 0.000444, FLG2-Elephant_00000160: 0.059260): 0.164766, (FLG-Opossum_00000106: 0.461181, FLG2-Tasmaniandevil_00000168: 0.244192): 0.001459): 0.014822): 0.012030, (((((FLG-GuineaPig_00000092: 0.104274, FLG-Mouse_00000090: 0.549526): 0.031819, (FLG-Chimpanzee_00000084: 0.000001, (FLG-Human_00000081: 0.000001, FLG-Gorilla_00000082: 0.000001): 0.000001): 0.146515): 0.000938, FLG-Cat_00000096: 0.213312): 0.000290, FLG-Elephant_00000102: 0.221474): 0.297499, (FLG2-Tasmaniandevil_00000162: 0.012310, FLG2-Opossum_00000164: 0.119507): 0.235223): 0.074421): 0.030743): 0.328552, (RPTN-Platypus_00000298: 0.436291, (((((RPTN-Gorilla_00000234: 0.014272, (RPTN-Human_00000233: 0.000001, RPTN-Chimpanzee_00000236: 0.000001): 0.009982): 0.132650, (RPTN-Mouse_00000252: 0.061911, RPTN-Rat_00000254: 0.000001): 0.228536): 0.034794, (((RPTN-Cow_00000258: 0.023455, RPTN-Sheep_00000288: 0.012142): 0.109764, RPTN-Pig_00000286: 0.045747): 0.011461, (RPTN-Dog_00000260: 0.057191, RPTN-Cat_00000262: 0.067589): 0.000130): 0.009278): 0.000254, RPTN-Elephant_00000266: 0.124335): 0.219785, (RPTN-Tasmaniandevil_00000294: 0.186345, RPTN-Opossum_00000296: 0.071637): 0.075981): 0.106617): 0.084801): 0.231034, (FLG-AnoleLizard_00000110: 0.603813, (FLG-Turkey_00000112: 0.087944, FLG-Flycatcher_00000116: 0.342666): 0.341688): 0.036398): 0.000240): 0.498390): 0.757090);

TREE #1 as a cladogram:

(((S100B-Elephant_00000774, ((((S100B-Rat_00000790, S100B-Mouse_00000792) 177 , S100B-GuineaPig_00000756) 176 , S100B-Rabbit_00000772) 175 , ((S100B-Cow_00000764, S100B-Pig_00000782) 179 , (S100B-Dog_00000788, S100B-Cat_00000770) 180 ) 178 ) 174 ) 173 , (S100B-Tasmaniandevil_00000794, S100B-Platypus_00000796) 181 ) 172 , ((S100B-Turkey_00000800, S100B-Duck_00000808) 183 , S100B-Flycatcher_00000802) 182 , ((((S100G-Opossum_00000898, S100G-Tasmaniandevil_00000900) 187 , ((((S100G-Cow_00000862, S100G-Pig_00000864) 191 , (S100G-Cat_00000872, S100G-Dog_00000868) 192 ) 190 , (((S100G-GuineaPig_00000876, (S100G-Rat_00000880, S100G-Mouse_00000886) 196 ) 195 , S100G-Rabbit_00000878) 194 , S100G-Human_00000827) 193 ) 189 , S100G-Elephant_00000892) 188 ) 186 , (((((MRP-126-Greenseaturtle_00002085, ((((S100A8-Turkey_00000478, S100A8-Chicken_00000476) 205 , (S100A8-Duck_00000480, MRP-126-Goose_00002001) 206 ) 204 , (MRP-126-EmperorPenguin_00002077, ((MRP-126-BarnOwl_00002030, MRP-126-Eagle_00002015) 209 , ((S100A8-Flycatcher_00000474, MRP-126-Zebrafinch_00002029) 211 , MRP-126-Budgerigar_00002048) 210 ) 208 ) 207 ) 203 , (MRP-126-Alligator_00002081, MRP-126-Saltwatercrocodile_00002082) 212 ) 202 ) 201 , ((S100A12-Elephant_00000364, ((S100A12-Rabbit_00000366, (S100A12-Human_00000307, (S100A12-Gorilla_00000308, S100A12-Chimpanzee_00000310) 218 ) 217 ) 216 , ((S100A12-Cow_00000356, (S100A12-Sheep_00000358, S100A12-Pig_00000360) 221 ) 220 , (S100A12-Cat_00000370, S100A12-Dog_00000338) 222 ) 219 ) 215 ) 214 , (S100A9-Tasmaniandevil_00000558, S100A9-Opossum_00000560) 223 ) 213 ) 200 , (S100A8-Platypus_00000472, (((((S100A8-Human_00000391, S100A8-Gorilla_00000392) 229 , (S100A8-Rabbit_00000416, ((S100A8-Rat_00000424, S100A8-Mouse_00000428) 232 , S100A8-GuineaPig_00000436) 231 ) 230 ) 228 , ((S100A8-Cat_00000442, S100A8-Dog_00000452) 234 , (S100A8-Pig_00000456, (S100A8-Cow_00000438, S100A8-Sheep_00000448) 236 ) 235 ) 233 ) 227 , S100A8-Elephant_00000462) 226 , (S100A8-Tasmaniandevil_00000468, S100A8-Opossum_00000470) 237 ) 225 ) 224 ) 199 , ((S100A12-Opossum_00000380, S100A12-Tasmaniandevil_00000382) 239 , (((((S100A9-Sheep_00000534, S100A9-Cow_00000538) 244 , S100A9-Pig_00000542) 243 , (S100A9-Cat_00000540, S100A9-Dog_00000544) 245 ) 242 , (((S100A9-GuineaPig_00000508, (S100A9-Mouse_00000514, S100A9-Rat_00000516) 249 ) 248 , S100A9-Rabbit_00000506) 247 , (S100A9-Human_00000483, S100A9-Chimpanzee_00000486) 250 ) 246 ) 241 , S100A9-Elephant_00000526) 240 ) 238 ) 198 , ((((((S100A7-Eagle_00003007, S100A7-BarnOwl_00003010) 256 , (S100A7-Zebrafinch_00003027, S100A7-Budgerigar_00003002) 257 ) 255 , S100A7-EmperorPenguin_00003023) 254 , ((S100A7-Goose_00003015, S100A7-Duck_00003021) 259 , S100A12-Turkey_00000386) 258 ) 253 , (S100A7-Saltwatercrocodile_00003038, S100A7-Alligator_00003036) 260 ) 252 , (((((S100A7-Cow_00000594, S100A7-Sheep_00000590) 265 , S100A7-Pig_00000608) 264 , (((S100A7-Chimpanzee_00000570, (S100A7-Human_00000569, (S100A7A-Chimpanzee_00000632, (S100A7A-Human_00000631, S100A7A-Gorilla_00000634) 271 ) 270 ) 269 ) 268 , (S100A7L2-Human_00000701, S100A7L2-Chimpanzee_00000702) 272 ) 267 , S100A7-Rabbit_00000622) 266 ) 263 , S100A7L2-Elephant_00000722) 262 , (S100A7-Tasmaniandevil_00000628, S100A7-Opossum_00000630) 273 ) 261 ) 251 ) 197 ) 185 , (((((((CRNN-Pig_00000046, (CRNN-Sheep_00000058, CRNN-Cow_00000062) 281 ) 280 , (CRNN-Cat_00000052, CRNN-Dog_00000044) 282 ) 279 , ((((CRNN-Mouse_00000030, CRNN-Rat_00000036) 286 , CRNN-GuineaPig_00000024) 285 , CRNN-Rabbit_00000038) 284 , (CRNN-Chimpanzee_00000002, (CRNN-Human_00000001, CRNN-Gorilla_00000008) 288 ) 287 ) 283 ) 278 , CRNN-Elephant_00000066) 277 , (CRNN-Opossum_00000072, CRNN-Tasmaniandevil_00000074) 289 ) 276 , (CRNN-Duck_00000080, (CRNN-Turkey_00000076, CRNN-Chicken_00000078) 291 ) 290 ) 275 , (((((HRNR-Cat_00000210, HRNR-Pig_00000206) 296 , ((HRNR-Gorilla_00000182, (HRNR-Human_00000179, HRNR-Chimpanzee_00000180) 299 ) 298 , (HRNR-GuineaPig_00000196, (HRNR-Rat_00000192, HRNR-Mouse_00000194) 301 ) 300 ) 297 ) 295 , ((FLG-Platypus_00000108, (((((FLG2-Rabbit_00000132, ((FLG2-Rat_00000138, FLG2-Mouse_00000140) 310 , FLG2-GuineaPig_00000150) 309 ) 308 , (FLG2-Human_00000117, FLG2-Chimpanzee_00000122) 311 ) 307 , (((FLG2-Sheep_00000134, FLG2-Cow_00000136) 314 , FLG2-Pig_00000146) 313 , FLG2-Cat_00000148) 312 ) 306 , FLG2-Elephant_00000160) 305 , (FLG-Opossum_00000106, FLG2-Tasmaniandevil_00000168) 315 ) 304 ) 303 , (((((FLG-GuineaPig_00000092, FLG-Mouse_00000090) 320 , (FLG-Chimpanzee_00000084, (FLG-Human_00000081, FLG-Gorilla_00000082) 322 ) 321 ) 319 , FLG-Cat_00000096) 318 , FLG-Elephant_00000102) 317 , (FLG2-Tasmaniandevil_00000162, FLG2-Opossum_00000164) 323 ) 316 ) 302 ) 294 , (RPTN-Platypus_00000298, (((((RPTN-Gorilla_00000234, (RPTN-Human_00000233, RPTN-Chimpanzee_00000236) 330 ) 329 , (RPTN-Mouse_00000252, RPTN-Rat_00000254) 331 ) 328 , (((RPTN-Cow_00000258, RPTN-Sheep_00000288) 334 , RPTN-Pig_00000286) 333 , (RPTN-Dog_00000260, RPTN-Cat_00000262) 335 ) 332 ) 327 , RPTN-Elephant_00000266) 326 , (RPTN-Tasmaniandevil_00000294, RPTN-Opossum_00000296) 336 ) 325 ) 324 ) 293 , (FLG-AnoleLizard_00000110, (FLG-Turkey_00000112, FLG-Flycatcher_00000116) 338 ) 337 ) 292 ) 274 ) 184 ) 171 ;
